# Perinatal treatment of parents with the broad-spectrum antibiotic enrofloxacin aggravates contact sensitivity in adult offspring mice

**DOI:** 10.1007/s43440-021-00217-3

**Published:** 2021-01-22

**Authors:** Paulina Kowalczyk, Anna Strzępa, Marian Szczepanik

**Affiliations:** Department of Medical Biology, Faculty of Health Sciences, Jagiellonian University Medical College, ul. Kopernika 7a, 31-034 Kraków, Poland

**Keywords:** Enrofloxacin, Antibiotic, Dysbiosis, Contact sensitivity, Immunomodulation

## Abstract

**Background:**

Antibiotics, while eliminating pathogens, also partially deplete commensal bacteria. Antibiotic-induced dysbiosis may contribute to the observed rise in “immune-mediated” diseases, including autoimmunity and allergy. The aim of this study is to investigate the impact of perinatal antibiotic treatment on T cell-mediated immune response in adult mice.

**Methods:**

Oral treatment with broad-spectrum antibiotic enrofloxacin during gestation and breastfeeding or breastfeeding or gestation alone was used to evaluate whether antibiotic exposure early in life could modulate contact sensitivity (CS) in adult mice.

**Results:**

Here, we demonstrated that enrofloxacin treatment during gestation and breastfeeding, but not during pregnancy or breastfeeding alone, aggravated CS reaction in adult mice measured by ear swelling. These data correlate with increased myeloperoxidase (MPO) activity in the ear extracts and elevated production of IL-6 and IL-17A by auricular lymph node cells (ELNC) and was not influenced by food consumption and body weight. In each dosing regimen, enrofloxacin treatment reduced the relative abundance of *Enterococcus* spp. but did not influence the relative abundances of *Lactobacillus*, *Clostridium* cluster XIVa, XIVab, I, *Bacteroidetes,* and segmented filamentous bacteria (SFB). However, prolonged enrofloxacin-treatment during both gestation and breastfeeding decreased the relative abundance of *Clostridium* cluster IV.

**Conclusion:**

These data show that long-term perinatal enrofloxacin treatment induces intestinal dysbiosis, characterized by decreased levels of anti-inflammatory *Clostridium* cluster IV, and alters T cell-dependent immune responses, enhancing CS reaction in adult mice.

## Introduction

Human microbiota resides on the skin and the mucosal surfaces of the respiratory, reproductive, and gastrointestinal systems, with the former exhibiting the most abundant community [1, 2]. The studies show that the fetus and the placenta are colonized as well [2, 3]. Alteration in the intestinal microbiota composition is observed in many autoimmune and allergic diseases and is described as “dysbiosis” [2]. On the other hand, targeted modification of the intestinal microbes could ameliorate the severity of immune-mediated disorders [4, 5].

Human microbiota supports the maturation of the infant immune response towards a Th1-type [1, 5]. Increased sanitation and antibiotic use have reduced our exposure to different types of antigens, referred to as the “microbial deprivation signal hypothesis,” and is potentially responsible for the observed rise in autoimmune and allergy disorders [6].

The main culprit of dysbiosis observed in adults and infants are antibiotics, the most frequently prescribed medications [7]. Animal studies have shown that antibiotic administration increased the severity of collagen-induced arthritis [8], whereas their use reduced the severity of experimental autoimmune encephalomyelitis [9] and allergic contact dermatitis [1]. Importantly, antibiotics are the most commonly prescribed drugs for pediatric patients [10]. It was shown that antibiotic use early in life increases the incidence of atopic disorders [11]. Approximately 40% of women receive antibiotics during pregnancy and just after the delivery [12]. As antibiotics can promote a Th2 immune response [13], the children from mothers treated with antibiotics during the perinatal period had an increased incidence of atopic dermatitis, asthma, and hay fever [14].

Contact sensitivity (CS) is a mouse model of allergic contact dermatitis in humans, which is the most common occupational disease. It develops after exposure to low molecular weight substances called haptens. The reaction is T cell driven and is classified as a type IV hypersensitivity. We have recently shown that dysbiosis induced by 2 weeks of enrofloxacin treatment reduced the severity of CS in adult mice by supporting the induction of cells with regulatory properties [1, 15]. The aim of the current study is to evaluate the impact of perinatal antibiotic use on the development of CS in the offspring mice.

## Materials and methods

### Mice

BALB/c mice were obtained from the breeding unit of the Department of Medical Biology, Jagiellonian University, College of Medicine. Mice were maintained under specific pathogen-free (SPF) conditions in individually ventilated cages and fed autoclaved food and water ad libitum. The experiments were approved by the 1st Local Ethical Committee on Animal Testing in Krakow (approval no. 35/2016).

### Treatment with enrofloxacin

**Fig. 1:**
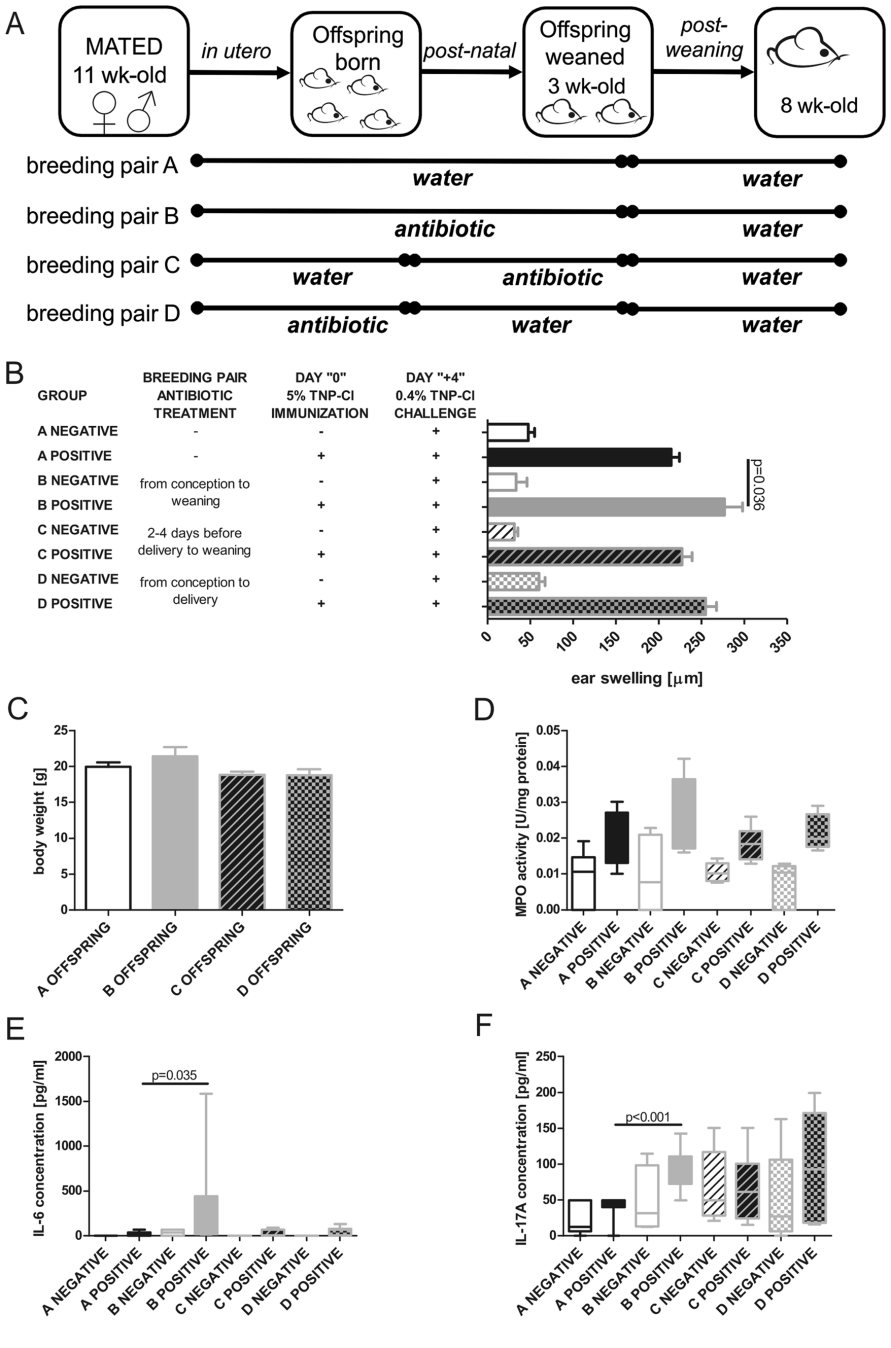
Prolonged perinatal treatment of the breeding pairs with the broad-spectrum antibiotic enrofloxacin aggravates CS in adult offspring. Breeding pairs were treated with enrofloxacin from conception to weaning (group B) or from 2 to 4 days before delivery until weaning (group C) or from conception until delivery (group D). Control breeding pairs received water alone (group A). At 8 weeks of age, the offspring from each breeding pair were immunized with sham treatment (negative) or 5% TNP-Cl (positive) to the shaved abdomen and chest. Four days later, all groups were ear challenged with 0.4% TNP-Cl, followed by a 24 h CS test. **a** Experimental design, **b** CS response (ear swelling) is aggravated in adult offspring from breeding pair B which was exposed to enrofloxacin from conception to weaning (mean ± SEM; *F*_7,66_ = 85.61, *p* = 0.000, *p* value at the graph for Tukey’s post hoc test), **c** enrofloxacin treatment does not change body weight (mean ± SEM), **d** MPO activity in ear homogenates (median with range), **e** concentrations of IL-6 (median with range; *U* = 22.5, *N*_A POSITIVE_ = 10, *N*_B POSITIVE_ = 10), and **f** IL-17A (median with range; *U* = 5, *N*_A POSITIVE_ = 10, *N*_B POSITIVE_ = 10) in ELNC culture supernatants. In case of **b** and **c**, statistical one-way ANOVA with Tukey’s post hoc test for unbalanced data was used. In case of **d**–**f**, between-group differences were calculated by the Mann–Whitney *U* test. *n* = 4–14

In this experiment, each breeder pair was set up by mating one male with one female. The breeder pairs were housed together through pregnancy and breastfeeding until weaning at the age of 3 weeks. Breeder pairs received drinking water containing the broad-spectrum antibiotic enrofloxacin (0.27 mg/ml). Breeder pairs were divided into four groups (A–D; Fig. 1a): group A received water only (control group), group B received antibiotic from conception to weaning, group C received antibiotic from 2 to 4 days before delivery until weaning, while group D received antibiotic from conception to delivery. On average, each group of breeder pairs had 6–7 offspring. The offspring of the breeder pairs exposed to enrofloxacin or water alone were subsequently studied for gut microbiota composition at the age of 3 weeks and CS response at 8 weeks of age.

### Sensitization and elicitation of contact sensitivity (CS) in vivo

For CS experiments, offspring of both sexes of breeder pairs receiving enrofloxacin or water were used. Mice were sensitized by skin application of 0.15 ml of 5% TNP-Cl in the acetone–ethanol mixture (1:3) to the shaved abdomen and chest. Control mice were treated with the acetone–ethanol mixture alone. Four days later, animals were challenged on both sides of the ears with 10 µl of 0.4% TNP-Cl in the olive oil–acetone mixture (1:1). The ear thickness was measured prior to testing with a micrometer (Mitutoyo, Tokyo, Japan) by an observer unaware of the experimental groups and then again at 24 h after the challenge. Ear thickness was calculated as [ear thickness (μm) 24 h after challenge – ear thickness (μm) before challenge] [1].

### Myeloperoxidase (MPO) assay

Neutrophil infiltration to the inflamed ears was indirectly quantitated using a MPO assay, as described previously [1]. Briefly, ears were removed 24 h post-challenge, and a 6-mm-diameter punch of the ear was excised, homogenized, freeze–thawed three times, followed by measurement of MPO activity. MPO activity was expressed in units per protein concentration (U/mg of protein).

### In vitro measurement of IL-6 and IL-17A in culture supernatants

To determine local production of cytokines in elicited CS, auricular lymph nodes (ELNs) were removed 24 h post-challenge. ELN cells (ELNC) were isolated and cultured (3 × 10^6^/ml) with 100 µg TNP_28_-Ig antigen (mouse immunoglobulins conjugated with TNP) in 1 ml complete RPMI 1640 medium. After 48 h, the culture supernatants were tested for cytokine concentration using an ELISA Set BD OptEIA ELISA Sets (BD Biosciences, San Diego, CA).

### Extraction of bacterial DNA from the gut content

Bacterial DNA from the feces was collected from the offspring of antibiotic-treated breeding pairs at the time of weaning (3 weeks after delivery), as described previously [1].

### PCR conditions

To evaluate dysbiosis, RT-PCR was performed with 10.5 ng of DNA using CFX96 Touch as described previously [1]. Detection of selected gut bacterial species and groups was based on the amplification of the conserved 16S rDNA sequences.

### Statistical analysis

Statistical calculations were performed using GraphPad Prism (GraphPad Software Inc, San Diego, CA, USA). The distribution of data was analyzed by the Kolmogorov–Smirnov test. Results are expressed as mean ± standard error of mean (SEM) or median with range. As the assumption of equal variance and normal distribution was retained, CS and body weight data were analyzed using the one-way ANOVA followed by Tukey’s multiple comparison test for unbalanced data. In vitro*,* between-group differences were calculated by the Mann–Whitney *U* test, as data were not normally distributed. The results were considered statistically significant when *p* < 0.05.

## Results

### Oral application of the broad-spectrum antibiotic enrofloxacin early in life aggravates CS in mice

Oral treatment of breeding pairs with enrofloxacin from conception to weaning aggravated CS in the offspring of both sexes, as measured by ear swelling (Fig. 1b). Treatment of breeding pairs with the antibiotic 2–4 days before delivery until weaning, or from conception to delivery, did not significantly affect CS susceptibility in the offspring (Fig. 1b; *F*_7,66_ = 85,61, *p* = 0.000, a post hoc Tukey test showed that group A positive and B positive differed significantly at *p* = 0.036). Treatment with enrofloxacin did not affect body weight in any group (Fig. 1c). However, a trend towards increased myeloperoxidase (MPO) activity in the ear extracts of the offspring from breeding pairs exposed to enrofloxacin from conception to weaning was observed (Fig. 1d). Additionally, in vitro culture of ELNC collected 24 h post-challenge showed a statistically significant increase of IL-6 (Fig. 1e; *U* = 22,5, N_A POSITIVE_ = 10, N_B POSITIVE_ = 10, *p* = 0.035) and IL-17A (Fig. 1f; *U* = 5, *N*_A POSITIVE_ = 10, N_B POSITIVE_ = 10, *p* < 0.001) production by ELNC from the offspring of breeding pairs exposed to enrofloxacin from conception to weaning.

### Oral treatment with enrofloxacin early in life modifies gut microbiota composition

**Fig. 2:**
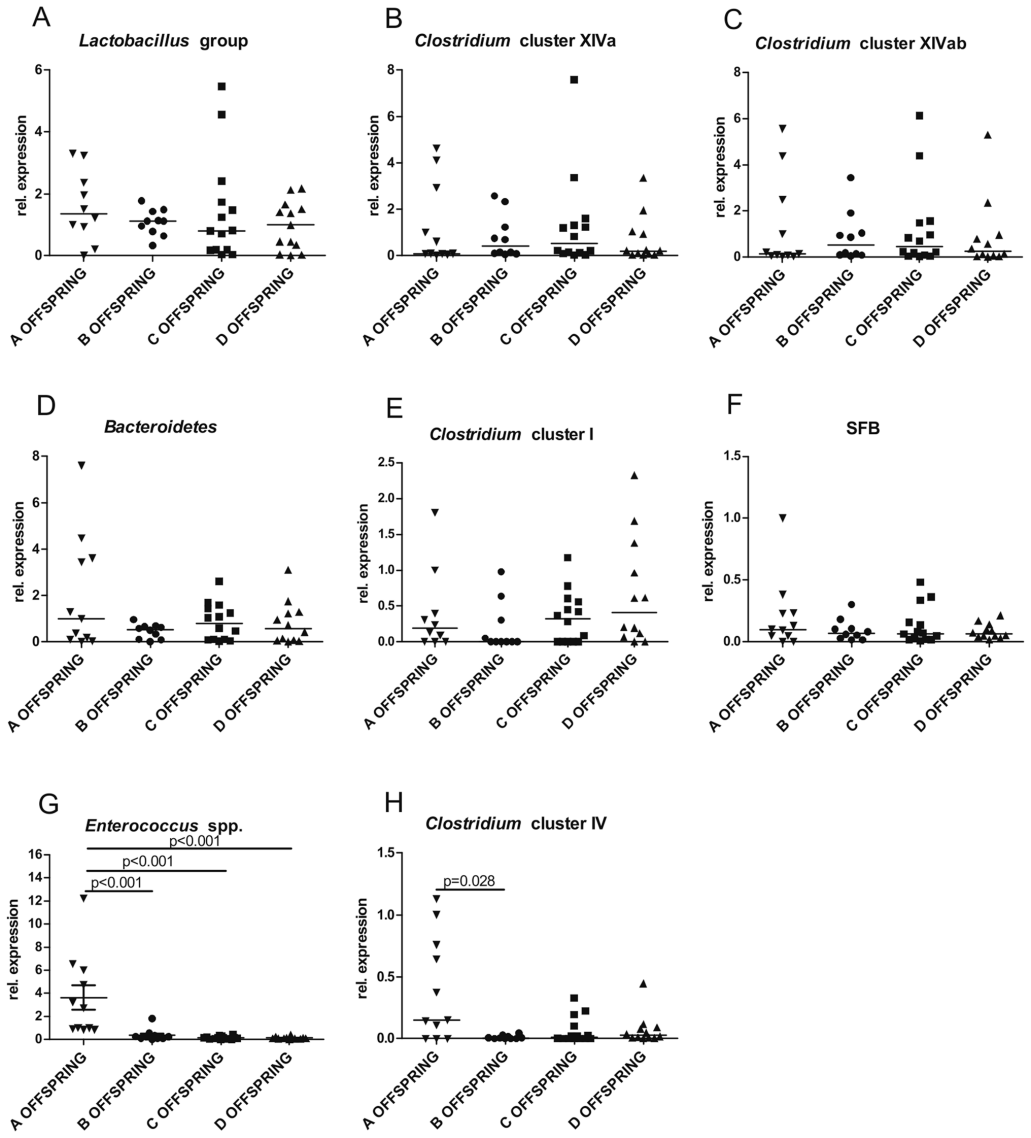
Prolonged perinatal treatment of the breeding pairs with broad-spectrum antibiotic enrofloxacin induces pro-inflammatory intestinal dysbiosis in the offspring at the time of weaning. Breeding pairs were treated with enrofloxacin in drinking water: from conception until weaning (group B), from 2–4 days before delivery until weaning (group C), from conception until delivery of the offspring (group D). Control breeding pairs received water alone (group A). At weaning (3 weeks of age), feces were collected from the offspring, and bacterial composition was investigated. Relative abundances of the highly conserved bacterial 16S rDNA fragments in feces from the following bacteria were analyzed **a** *Lactobacillus*, **b** *Clostridium coccoide*s (cluster XIVa), **c** *Clostridium coccoides*–*E. rectale* (cluster XIVab), **d** *Bacteroidetes,* **e** *Clostridium* (cluster I), **f** SFB, **g** *Enterococcus spp*., (*U*_AB_ = 5, *U*_AC_ = 0, *U*_AD_ = 0, *N*_A_ = 11, *N*_B_ = 10, *N*_C_ = 14, *N*_D_ = 12), **h** *Clostridium* cluster IV (*U*_AB_ = 24, *N*_A_ = 11, *N*_B_ = 10). Between-group differences were calculated by the Mann–Whitney *U* test. Results are shown as median

Many studies indicate that the peripheral immune response is regulated by gut bacteria, promoting either anti-inflammatory or pro-inflammatory properties. We identified an increased CS response in the offspring of breeder pairs treated with enrofloxacin from conception to weaning. To test if this resulted from altered microbial colonization of the offspring, the composition of pro- or anti-inflammatory intestinal bacteria of the offspring using RT-PCR at the time of weaning (3 weeks after delivery) was evaluated. Oral treatment with enrofloxacin from conception to weaning (Offspring B), from 2 to 4 days before delivery until weaning (Offspring C) or from mating to delivery (OFFSPRING D) did not influence the relative abundance of *Lactobacillus* (Fig. 2a), *Clostridium coccoide*s (cluster XIVa) (Fig. 2b), *Clostridium coccoides*–*E. rectale* (cluster XIVab) (Fig. 2c), *Bacteroidetes* (Fig. 2d)*, Clostridium perfringens* (cluster I) (Fig. 2e) and segmented filamentous bacteria (SFB) (Fig. 2f). However, oral treatment with enrofloxacin significantly reduced the relative abundance of the common gut commensal *Enterococcus spp*. (Fig. 2g; *U*_AB_ = 5, *U*_AC_ = 0, *U*_AD_ = 0, *N*_A_ = 11, *N*_B_ = 10, *N*_C_ = 14, *N*_D_ = 12, *p*_AB_ < 0.001, *p*_AC_ < 0.001, *p*_AD_ < 0.001) in each group regardless of the time enrofloxacin treatment was administered. Interestingly, enrofloxacin treatment decreased the relative abundance of *Clostridium* cluster IV (Fig. 2h, *U*_AB_ = 24, *N*_A_ = 11, *N*_B_ = 10, *p*_AB_ = 0.028) only in the offspring of the breeder pairs treated with enrofloxacin from conception to weaning compared to control mice born from breeder pairs receiving water only.

## Discussion

In this study, we evaluated the influence of perinatal antibiotic treatment on the development of allergic contact dermatitis using CS to TNP-CL as a model. Here, we demonstrated that prolonged perinatal enrofloxacin administration from conception till weaning influences the developing immune system, resulting in aggravated CS at 8 weeks of age post-immunization and challenge.

Aggravated CS response correlated with increased MPO activity in ear tissue and increased IL-6 and IL-17A production by ELNC. However, time-restricted enrofloxacin treatment of breeding pairs, referring to either enrofloxacin administration 2–4 days before delivery until weaning or preconception to delivery, did not influence the severity of CS response in the offspring.

Intestinal microbiota regulates inflammatory responses in numerous diseases. Patients suffering from Th1/Th17-cell-driven rheumatoid arthritis, multiple sclerosis, Crohn's disease (CD), or from Th2-driven pathologies such as atopic dermatitis and asthma have altered gut microbiota compositions [2]. Dysbiosis is also observed in patients with early symptoms [16], implying that the gut microbiota influence early immune changes. This association is well seen in germ-free (GF) mice that are devoid of any microorganisms, as reduced severity of collagen-induced arthritis (CIA) and experimental autoimmune encephalomyelitis (EAE). On the other hand, their allergic responses to food antigens or airborne ovalbumin (OVA) are aggravated [2]. The altered reactivity of the immune system in GF mice is a consequence of an immature immune system, characterized by elevated Th2 immune response compared to SPF mice, implying that intestinal microbiota is important for the maturation of the immune system [2, 17]. Antibiotics are one of the most important factors that modify intestinal microbiota composition, causing improper maturation and altered immunoreactivity.

In this study, we used the broad-spectrum antibiotic enrofloxacin. Antibiotics are the most common medication used by adults and children and are often given prenatally [7, 10, 12]. Most newborns are exposed to antibiotics at some point during the perinatal period. Prior to delivery, antibiotic exposure may arise from cesarean prophylaxis or in the prevention of neonatal sepsis. Additionally, many women receive antibiotics during pregnancy to treat respiratory or genital infections and bacterial vaginosis [12]. Thus, the common use of antibiotics prenatally is a crucial factor in contributing to the disruption of the immune system maturation and molding the immune reactivity in newborns. Alteration of the immune system in newborns could also arise as a consequence of pharmacotherapy with antiepileptic drugs during pregnancy and breastfeeding [18, 19]. This class of drugs was shown to mediate epigenetic changes that could lead to “immune response imprinting” [20, 21].

Here, we have demonstrated that the offspring of breeding pairs exposed to antibiotics throughout pregnancy and breastfeeding develop aggravated CS at the 8 weeks of age post-immunization and challenge. We have previously shown that enrofloxacin treatment ameliorated the severity of CS in adult mice. Two-week enrofloxacin treatment-induced intestinal dysbiosis is characterized by increased abundances of anti-inflammatory but decreased abundances of pro-inflammatory bacteria [1]. Enrofloxacin-induced dysbiosis generated an anti-inflammatory environment characterized by the increased secretion of TGF-*β* and IL-10, locally in the Peyer’s patches and systematically in the spleen. This anti-inflammatory environment was associated with an increased proportion of tolerogenic dendritic cells, which are known inducers of Treg cells [15]. Additionally, enrofloxacin-induced dysbiosis supported the development of a broad spectrum of cells with regulatory potential, including TCRαβ^+^CD4^+^CD25^+^FoxP3^+^ Tregs, CD19^+^B220^+^CD5^+^IL-10^+^ B cells, IL-10^+^ Tr1 cells, and IL-10^+^TCRγδ^+^ cells, that efficiently suppressed the CS response [1], which correlates well with other studies. Application of antibiotic cocktails or broad-spectrum antibiotics to adult mice reduced the severity of Th1-dependent EAE [9, 22] and Th17-dependent psoriasis [23]. Antibiotic treatment was also an effective strategy to reduce the severity of symptoms in RA patients [2], although enrofloxacin treated mice have aggravated CIA [8].

Contrary to our previous study, where enrofloxacin treatment of adult mice led to amelioration of the CS reaction, here, we observed that perinatal enrofloxacin treatment of breeding pairs aggravates the CS response in the offspring at 8 weeks of age as evident by increased ear swelling. A limited number of studies show that perinatal antibiotic treatment of parents aggravates the severity of Th1/Th17 and Th2-dependent pathologies in adult offspring. Offspring of mice treated with an antibiotic cocktail of vancomycin and polymyxin B for 3 weeks after delivery develop aggravated Th17-dependent psoriasis [23]. Similarly, enrofloxacin treatment provided by breastfeeding aggravates Th2-dependent response to OVA when applied intranasally [24] or transdermally [13]. These data are in line with the “microbial deprivation signal hypothesis” which states that reduced exposure, or lack of bacterial signals, leads to rising autoimmunity and allergy incidences, which continue to be observed at present [6].

Interestingly, we did not observe a statistically significant increase in the CS response in adult offspring when enrofloxacin was administered to breeding pairs either 2–4 days before delivery to weaning or from conception to delivery. However, enrofloxacin treatment during breastfeeding is sufficient to modulate Th2-dependent pathologies in adult offspring [13]. Also, increasing the number of antibiotic courses, leading to long-term antibiotic exposure, results in a higher incidence of allergic diseases [14]. These data, as well as our own, imply that the short-term perinatal enrofloxacin treatment of the parents is sufficient to aggravate Th2 but not Th1/Th-17 CS response in the adult offspring. Short-term enrofloxacin treatment of the parents can have a variable impact on Th1/Th17 and Th2 immune responses in the adult offspring, which appears to be dependent on the degree to which the microbiota composition is altered and how well the immune system is stimulated by the remaining microbiota.

Since microbial signals shape and regulate the maturation of the immune response, we evaluated the composition of bacteria in the feces of the offspring at weaning. Both short- and long-term enrofloxacin treatment of breeding pairs was sufficient to decrease the relative abundance of the common gut commensal *Enterococcus spp*. but it did not affect the relative abundances of *Lactobacillus*, *Clostridium coccoides* (cluster XIVa), *Clostridium coccoides–E. rectale* (cluster XIVab), *Bacteroidetes*, *Clostridium* (cluster I), and SFB, in the feces of the offspring at weaning. However, long-term enrofloxacin treatment decreased the relative abundance of *Clostridium* cluster IV in the feces of the offspring at weaning. Bacteria belonging to *Clostridium* cluster IV have strong anti-inflammatory properties as they increase the concentration of TGF-β in the intestines and support the development of IL-10-producing inducible Treg cells [25]. *Clostridium* cluster IV bacteria produce short-chain fatty acids (SCFA) [26], which are potent inducers and activators of Treg cells [2]. Inoculation of 2 week old neonatal SPF mice with *Clostridium* cluster IV-containing feces increased the number of Tregs in the intestines and reduced the severity of dextran sodium sulfate (DSS)-mediated colitis, and Th2-dependent oxazolone-induced colitis [25]. Consistent with these data, we found a reduced abundance of anti-inflammatory *Clostridium* cluster IV bacteria in the offspring of the breeding pairs treated with enrofloxacin throughout pregnancy and during breastfeeding that could explain increased CS response in offspring at 8 weeks of age. Altered microbial colonization of the offspring could have not only maternal but also paternal origin. It was shown previously that the paternal preconception diet changes the gut bacterial composition in the offspring [27]. Also, the seminal fluid microbiome could change the health of the offspring [28].

In summary, this data indicates that prolonged perinatal antibiotic treatment of breeding pairs promotes T cell-mediated pathologies in the adult offspring by inducing intestinal dysbiosis at weaning. Altered microbiota composition, characterized by the decreased level of *Clostridium* cluster IV, may directly or indirectly (through SCFAs) alter the inflammatory response and the subsequent development of the neonatal immune system. Additional work is required to investigate further the influence of microbiota modification early in life in mediating susceptibility to the CS responses.

## Abbreviations

CIA: Collagen-induced arthritis
CS: Contact sensitivity
EAE: Experimental autoimmune encephalomyelitis
ELNC: Auricular lymph node cells
GF: Germ free
IL: Interleukin
MPO: Myeloperoxidase
OVA: Ovalbumin
PBS: Phosphate-buffered saline
RT-PCR: Reverse transcription-polymerase chain reaction
SCFA: Short-chain fatty acids
SFB: Segmented filamentous bacteria
Th: Helper T lymphocytes
TNP-Cl: Trinitrophenyl chloride
